# Aspects of sustainability and design engineering for the production of interconnected smart food packaging

**DOI:** 10.1371/journal.pone.0216555

**Published:** 2019-05-08

**Authors:** María Inés Cabot, Amalia Luque, Ana de las Heras, Francisco Aguayo

**Affiliations:** Department of Design Engineering, Higher Polytechnic School, University of Seville, Seville, Spain; Central Agricultural University, INDIA

## Abstract

In the present work, the problem of food wastage and the concept of sustainability are studied. An analysis of Life Cycle Assessment as a tool and of the innovative concept of Cradle to Cradle is also carried out, together with an exhaustive comparison of these two approaches. Based on these concepts, an integrated methodology is proposed for the design of interconnected smart products. The smart packaging systems currently available are studied theoretically and a practical case is analysed using the proposed methodology through the design and Life Cycle Assessment of a smart interconnected container that is able to detect the ethylene emitted by climacteric fruit, thereby minimizing food wastage. For the case under study, a major impact is observed of the selected plastics in the resources category, and of the smart system in the human health category.

## Introduction

The objectives of the present work include research into various methodologies and innovative frameworks for the study of the sustainability of products and processes, specifically that of Life Cycle Assessment (LCA) and Cradle to Cradle (C2C). A comparison is made between these two approaches and a combined methodology is proposed, which then can be applied to the design of smart interconnected food packaging.

Smart Products are mechatronic products that are additionally equipped with embedded systems enabling communication with other Smart Products using existing internet technologies [1,2].

The current definition of sustainability dates from 1987: "Sustainability or sustainable development is one that meets the needs of the present without compromising the needs of future generations"[3–5]. This encompasses three major stages that define the 3E strategy: economy, equity, and ecology, constituting the Triple Bottom Line [6], where the three dimensions are treated equally [7,8].

The last two stages in the history of sustainability involve eco-efficiency ("from cradle to grave") [9,10], which covers all stages of the life cycle, from the extraction of materials to the end of life of the products, and the eco-effectivity ("from cradle to cradle") [11–13], whose objective is not only to assure the efficiency of the processes throughout the stages of the life cycle, but also to ensure that, at the end of the useful life of products, their materials can be reused or recycled [14].

The whole value chain of a product has the responsibility to explain that sustainability is not synonymous to recycling, recyclability, recycled content, biodegradability and other buzzwords, but that the efficiency of the supply chain resources should remain the top priority[15].

This is why any improvement to sustainability requires the knowledge of complete value chains, and a focus on a single section is insufficient since solving a problem in one place in the chain can result in the creation of a different problem in another part of the chain [16].

According to the FAO [17], "food waste" can be defined as food suitable for human consumption that is discarded as a result of negligence or a conscious decision to throw away food. The earlier a product is lost or wasted in the supply chain, the higher the environmental cost becomes, and hence preventing the avoidable generation of food waste in the supply chain represents the most advantageous option within the hierarchy of food wastage [18].

The design of the food container constitutes one of the principal causes of food waste [19], since many times food packages are difficult to empty. In addition to this consideration, they should also be easy to reseal in order to avoid biological deterioration, be provided in sizes that obviate food being left over, and supply the correct information (content, expiration date, etc.) in order to prevent fresh food from being discarded [20]. New packaging solutions enable the economic aspect of food wastage to be improved and interest in active and smart packaging is currently on the rise. Field research has been carried out on packaging characteristics and technologies [21]. This is evidenced by the number of patents and patent applications granted in recent years [22].

The objective of the present case study is to study the environmental impacts of a smart interconnected rigid plastic container, whose main purpose is to store climacteric fruit in the refrigerator of a standard household. This container monitors the ethylene content emitted by the fruit during its ripening process and informs consumers via a Bluetooth connection that the fruit it contains has reached its optimum for consumption, thus preventing food from being wasted.

This container is designed to be transported from the house to the supermarket every time a purchase thereof is made, which implies a saving in the disposable plastic containers commonly used in the packaging of this type of product.

The rest of the paper is organized as follows: Section 1 deals with the concept of sustainability, food waste, and the importance of packaging for its minimization, and this section concludes with the explanation of the case study. In Section 2, which corresponds to materials and methods, Life Cycle Assessment Methodology and Cradle to Cradle Philosophy are studied, the Smart Packaging concept is analysed, the MOSFET Sensors are chosen as the appropriate smart technology, and the choice of plastics for the manufacture of the container is explained.`

Metal-oxide semiconductor field-effect transistor (MOSFET) sensors rely on a change of electrostatic potential. A MOSFET sensor comprises three layers: a silicon semiconductor; a silicon-oxide insulator; and a catalytic metal (usually palladium, platinum, iridium, or rhodium), also called the gate [23]. A MOSFET requires a thin semiconducting layer, which is separated from a gate electrode by the gate oxide dielectric, and also requires source and drain electrodes of width W (channel width) separated by a distance L (channel length) [24].

In Section 3, where the theory and calculations are discussed, the objective and scope are outlined and the inventory of mass and energy is analysed. The end of life of the packaging is then discussed. Section 4 presents the results of the comparison between the approaches of Life Cycle Assessment and Cradle to Cradle in terms of impact characterization, evaluation by standardized category, and single score by component, obtained from SimaPro software. In the subsequent discussion section (Section 5), the basic discrepancies between the two methods, the measurement of C2C principles with LCA, and the application of C2C principles to the case study are all discussed, and a process optimization is carried out. Finally, Section 6 presents the conclusions.

## Materials and methods

### Methodology of Life Cycle Assessment (LCA)

Life Cycle Assessment is a methodology developed in the 1970s to measure the impact of a product, service, or process throughout its life cycle, from the time its raw materials are obtained to their end of life and subsequent management [25].

Based on the collection and analysis of inputs and outputs of the system (natural resources, emissions, waste, and by-products) to obtain quantitative data on their potential impacts on the environment [25], LCA considers that all the stages involved in the life cycle of a product/activity are responsible for its environmental consequences.

Certain studies indicate that agricultural production constitutes the hotspot in the life cycle of food products, and that LCA can assist in identifying the most sustainable options [26].

The European Union has indicated that LCA is the best tool for the evaluation of the potential environmental impact of products [27].

**Fig 1:**
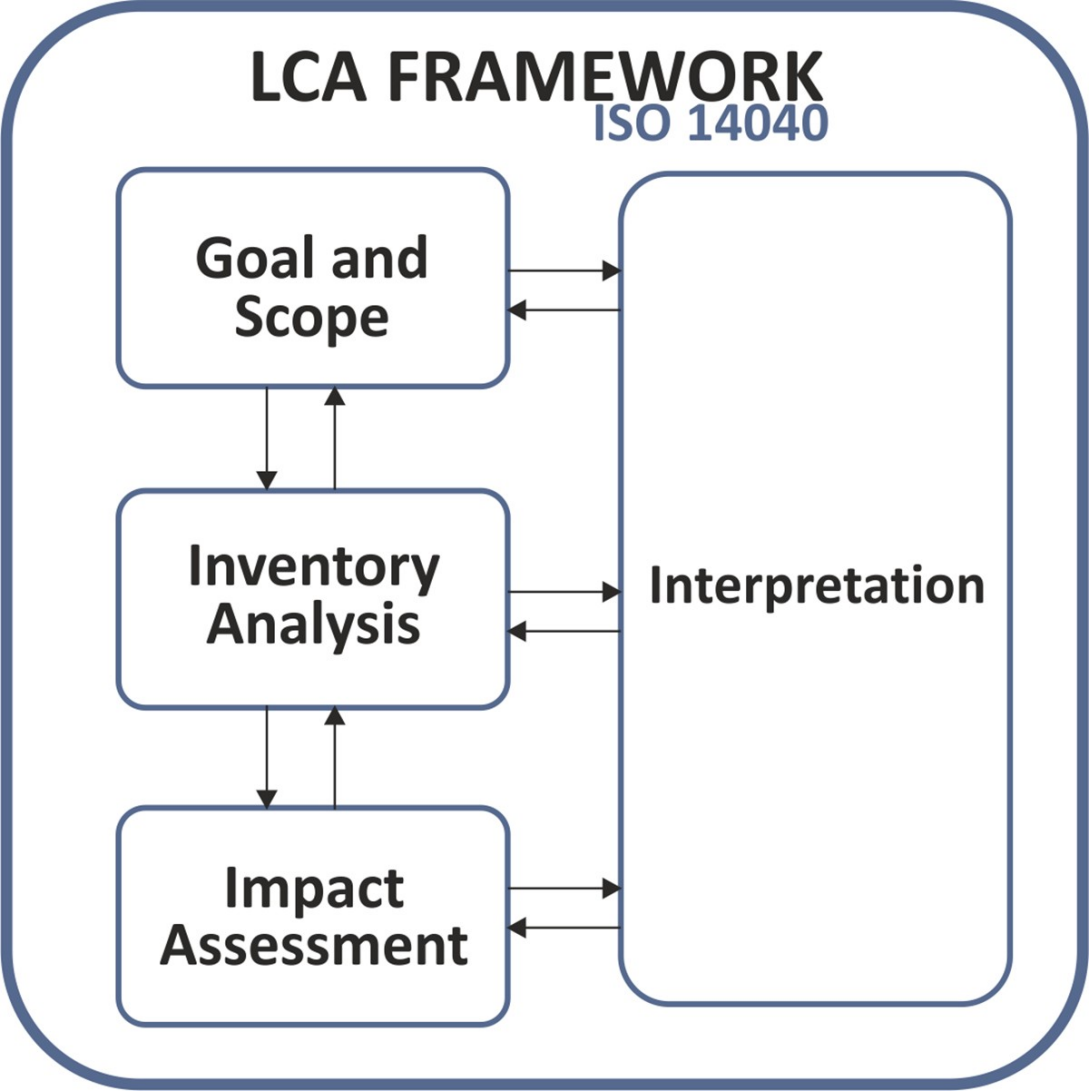
ISO 14040:2006. Adapted from [32].

The LCA methodology consists of 4 steps (Fig 1):

Step 1: Definition of objectives and scope [28–30];
Step 2: Inventory analysis [28,31];
Step 3: Impact evaluation [27];
Step 4: Interpretation of results [29].

The LCA method is dynamic, and the four stages in which it is performed are related to each other in such a way that, as results are obtained, then the data, hypotheses, system limits, and/or objectives, can all be modified or improved.

### Cradle to Cradle Philosophy (C2C)

Cradle to Cradle is an innovation framework used since the 1990s in order to design products and services that are beneficial in economic, well-being, and environmental terms, in order to achieve a sustainable world [33]. Cradle to Cradle (C2C) offers a positive vision of the future, where products are radically redesigned to be beneficial both to humans and the environment. This concept is not based on the reduction of negative impacts (as in LCA), but on the increase of positive impacts [13,34,35]. A transition towards a circular economy requires consumers and companies to shift towards models of utilisation rather than of ownership.

The C2C approach proposes replacing eco-efficiency with eco-effectivity, where the challenge is to make positive net contributions to the built environment with the aim of achieving "zero status”: zero residual emissions, zero use of resources, and zero toxicity, while taking up the banner of the declared goal of “doing good” instead of simply avoiding “doing much wrong” [36].

The basis of the C2C philosophy is that, although nothing good can be achieved by making small changes in a system that is fundamentally wrong, the bad things can still be reduced a little [37].

The aspiration of C2C for the built environment involves the promotion of smart designs that enjoy a positive synergic relationship with the environment. This can only be achieved by taking inspiration from natural flow systems where the sun is the main source of energy and where process waste is metabolized biologically to feed nourishment for other biological processes [33].

It is emphasized that the products should be designed as biological nutrients or technical nutrients, the former composing the naturesphere, which is metabolized and regenerated by nature, and the latter composing the technosphere, which is maintained in closed cycles of manufacture, reuse, and recovery [37].

This philosophy prioritizes supra-recycling, where of a material or product without use, destined to be waste, is transformed into another material or product of equal or greater utility or value, and the waterfall model, where the materials are kept within a technical cycle for a certain number of iterations, until losing their properties before returning to the biological cycle [13].

This framework raises 3 principles. The first is the concept of closed cycles, where the waste of one process constitutes the food of another process, thereby rejecting the eco-efficient approach of striving to reduce the amount of waste, and stating that the vision should be to design systems with products that other processes can absorb as nutrients [37]. This principle also incorporates the concept of toxicity of the material [33].

The second principle involves the use of sustainable energy and proposes that the energy demanded by industrial activity be obtained preferably from renewable sources, such as energy from the sun [33], instead of exploiting abiotic resources in the form of fossil fuels, which devastates regions where these materials have been stored [14].

The third principle is called "celebrating diversity" and is based on the fact that diversity favours the resilience and robustness of the product and the associated system, thereby guaranteeing security in a changing world [14]. Therefore, in order to improve the resistance of a system, diversity is necessary. Focusing on only one criterion could cause instability and imbalance in a broader context [13].

### Smart packaging systems

In legal terms, these are known as "intelligent materials and objects in contact with food" and can be defined as the materials and objects that control the state of packaged foods or their surroundings [38]. These include systems employed to detect, perceive, and record any changes within the package during its life cycle [39] and to communicate this information related to the quality or safety of the packaged product [18] in order not only to improve safety and quality, but also to warn about potential problems during the transport and storage of food [39].

Unlike active packaging, smart packaging does not intend to release components into the food [40].

The emergence of smart packaging systems has contributed towards a significant change in the existing perception of packaging, since they transform the traditional communication functions of packaging into intelligent communications [22], and interact with the consumer in terms of their ability to detect changes in the environment of the products [41].

Controlling the state of food content and alerting the consumer when food begins to deteriorate or is no longer fit for consumption has the potential to eliminate the waste caused by today's "use by" dates that are necessarily conservative. While the smart package may require more resources for its production, the food supply system it supports will become much more sustainable [16].

**Fig 2:**
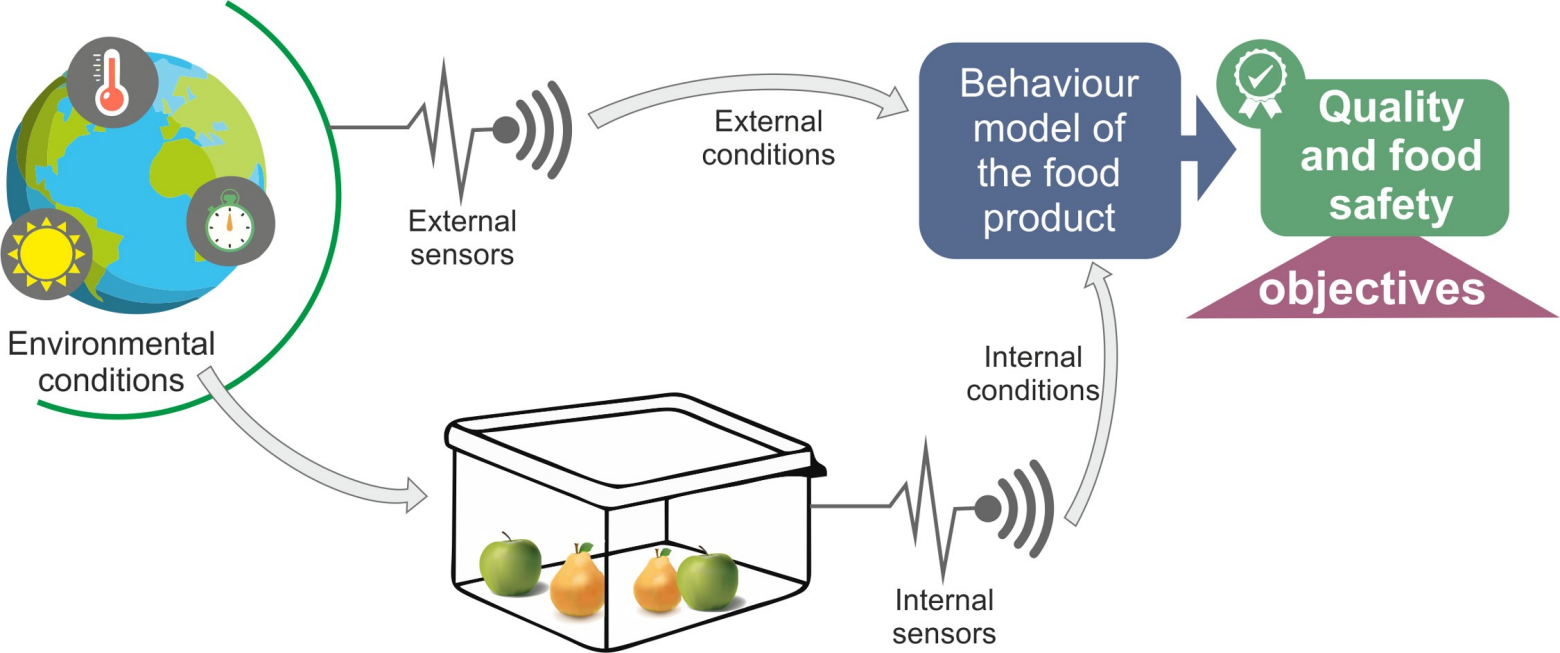
Product-packaging-environment interaction for smart packaging.

One concept of smart packaging includes a system of interactions between the product, the package, and the environment as can be observed in Fig 2.

If this technology were combined with low-impact packaging systems, there would be an increase in the environmental sustainability of the packaging and food preservation solution. Furthermore, if the contained food were produced using processes and products of low environmental impact, all the packaged food would be more sustainable [42].

### Intelligent technology: MOSFET sensors

A sensor can be defined as a device used to detect, locate, and/or quantify energy or matter that gives a signal for the detection or measurement of a physical or chemical property to which the device responds [41]. Chemical sensors generally contain two basic components connected in series: a molecular chemical recognition system (receptor) and a physicochemical transducer [43].

Receptors are based on the detector selectivity of the sensor, and can transform, in a continuous and reversible way, a chemical or physical parameter into energy, while a transducer converts this energy into a useful analytical signal, that is, an electrical signal.

In comparison with indicators, sensors are fast, accurate, and reliable, although their application in packaging systems is often more complicated. Indicators are more commercialized than sensors because they are more economical and can be verified at a glance; however, they fail to provide information on the quality of food in real time [39].

It is of special importance that the reaction with ethylene in the chosen sensor is reversible, since the container will be reused.

The technology chosen for the smart part of the container includes metal-oxide semiconductor field-effect transistor (MOSFET) sensors. These sensors comprise three layers: a silicon semiconductor, an insulator of silicon oxide, and a catalytic metal (Pd, Pt, Ir, Rh) called the "gate" [44].

An MSP430 microcontroller, connected to the electric current, is attached to the sensors.

### Plastics chosen for the manufacture of the container

The packaging is made of plastic since it is in this material that most innovations are made. There is also an addition of two plastic layers, so that the desired physical and barrier properties are achieved. The property of resistance is a major priority, since it holds the key to achieving the desired useful life and minimizes any damage in the transport of the food to the supermarket.

The use of bioplastics was considered, but these materials have less favourable barrier and mechanical properties than common plastics, and would result in an undesirable increase in the input of material. In addition, conversions involving energy consumption are required to move bioplastics from biological raw materials to useful molecules [16].

On the other hand, the use of biodegradable plastics was also considered; their useful life is approximately 6 months, during which they gradually lose their properties. Logically, since a container with a long shelf life is desired, this is not an option, at least initially.

Finally, the container is composed of an outer layer of Polyethylene Terephthalate (PET), with high hardness and low oxygen permeability and an internal layer of High Density Polyethylene (HDPE), which provides impact resistance and low moisture permeability. A mixture of 75/25 PET/HDPE is used, and as an adhesive, the copolymer commercially known as Surlyn (a copolymer of ethylene and 6.5% by weight of methacrylic acid partially neutralized with zinc) is chosen, since its operation has been observed in studies, such as [45].

## Theory and calculations

For the Life Cycle Assessment of the interconnected smart packaging, SimaPro Software and the Ecoindicator 99 methodology are used.

### Objective and scope

Life Cycle Assessments should be based on a defined service unit: the functional unit delivered to a consumer through the value chain. Therefore, for the present study, one (1) interconnected intelligent plastic PET/HDPE bilayer container with a volume of 5L was chosen as the functional unit [16].

The scope of the Life Cycle Assessment covered the transport and extrusion of PET and HDPE granules, the coextrusion of the laminates obtained, the thermoforming of the final packaging, the coupling of the sensors in the container, the distribution of the containers in the Spanish territory, the use of the container, and then the analysis of its possible disposal at the end of its useful life.

The Ecoindicator 99 methodology is used, and hence the scope of this LCA covers the following impacts: climate change, depletion of the ozone layer, acidification/eutrophication (combined), carcinogenesis, organic respiratory compounds, inorganic respiratory compounds, ionizing radiation, eco-toxicity, land use, mineral resources, and fossil resources.

### Inventory analysis

#### Mass inventory

The measures of the container are established under the consideration that it has to be transportable, and consistent with the purchases made by a family type, which is set at 2 Kg of climacteric fruit per week (12 medium apples), which corresponds to a volume of 4560 L, and that it should be rectangular in shape in order to be practical at the time of placing it inside the refrigerator.

The final measurements are determined: length 0.25m, width 0.20m, and height 0.10m, with a lid length of 0.25m, width 0.20m, and height 0.02m, thereby establishing a total volume of 5000L.

The thickness is set at 1cm of which considered a logical measure and approximately 0.0075m is PET and 0.0025m HDPE.

The masses of the sensor and the MSP430 microcontroller employed are both estimated at 0.05Kg.

The masses of PET, HDPE, and Surlyn are determined by the container measurements and the density of the materials, resulting in values of 2.15 kg of PET, 0.99 kg of HDPE and 0.24 kg of Surlyn per container.

#### Energy inventory

Three energy inputs are determined. The first is production: extrusion, coextrusion, and thermoforming are observed within this stage.

The second entry is that of use: the sensor is considered as turned on once every 6 hours, and this sensor determines the shelf life of the container itself, since it is in contact with the atmosphere of the food and is estimated to have greater use than the other components. Its useful life is 15 months.

The main elements of this stage include the MOSFET sensor, for which a measurement time of 10 min and a maximum current of 0.1 A are determined, leading to a calculated consumption of 24840000J, and the microcontroller MSP430, which in active mode consumes 0.00025A, and in real-time clock mode (when the sensor is not measuring) consumes 1e-6A, with a total consumption calculated for the 15 months at 62100J.

The third entry occurs during the distribution, for which trucks are chosen that transport 40 tons and comply with the Euro 5 standard. It is assumed that the acquisition of the inputs for the container, and their distribution is carried out in Spain. A distance of 500 km is therefore used, which constitutes the average distance between the main Spanish cities.

For this stage, post-consumer waste collection is excluded.

### Discussion of the end of life of the container

Multilayer plastic is not recyclable, and hence end-of-life scenarios may initially only include incineration and disposal to landfills [46]. On the other hand, PET does not degrade in landfills, and it has been shown that there is no degradation of organic compounds in landfills due to the presence of an anaerobic environment [47], therefore, this scenario is not an option for the container in this study either. The possible option for the smart packaging involves incineration, carried out with energy recovery.

## Results

### Life Cycle Assessment (LCA) and Cradle to Cradle (C2C) comparison

To begin with, C2C is a philosophy that establishes clear objectives at the beginning of the process, while LCA proposes the identification of "hotspots" together with the measurement of the environmental burden of a product [48].

The C2C approach enhances the creation of beneficial products, while in the LCA, products contaminate by definition, since they consume resources and finally produce some type of waste at the end of life [48].

Regarding intellectual property, in the case of C2C, the McDonough Braungart Design Chemistry (MBDC) [11] in Charlottesville, Virginia, owns the Cradle to Cradle trademark and the Environmental Protection Encouragement Agency (EPEA) in Hamburg, Germany holds the licence for its use. The C2C Product Innovation Institute (California) is in charge of the certification of the products.

On the other hand, the LCA has been developed by a global community of professionals and is not owned by anyone in particular. Part of its coordination is under the authority of the UNEP-SETAC Lifecycle, and it is ISO 14040/44 that defines the applicability framework [48].

In terms of communication and marketing, C2C places the client as part of the solution through the purchase of certified products, while the LCA communicates the hotspots that can be reduced through redesign [48].

The LCA can be used to compare different but functionally similar products while the C2C compares one product in the different stages of its optimization [48].

The C2C approach states that the best source depends on the local situation, while for the LCA the best source depends on the calculation of environmental impacts [48].

### Impact characterization

**Fig 3:**
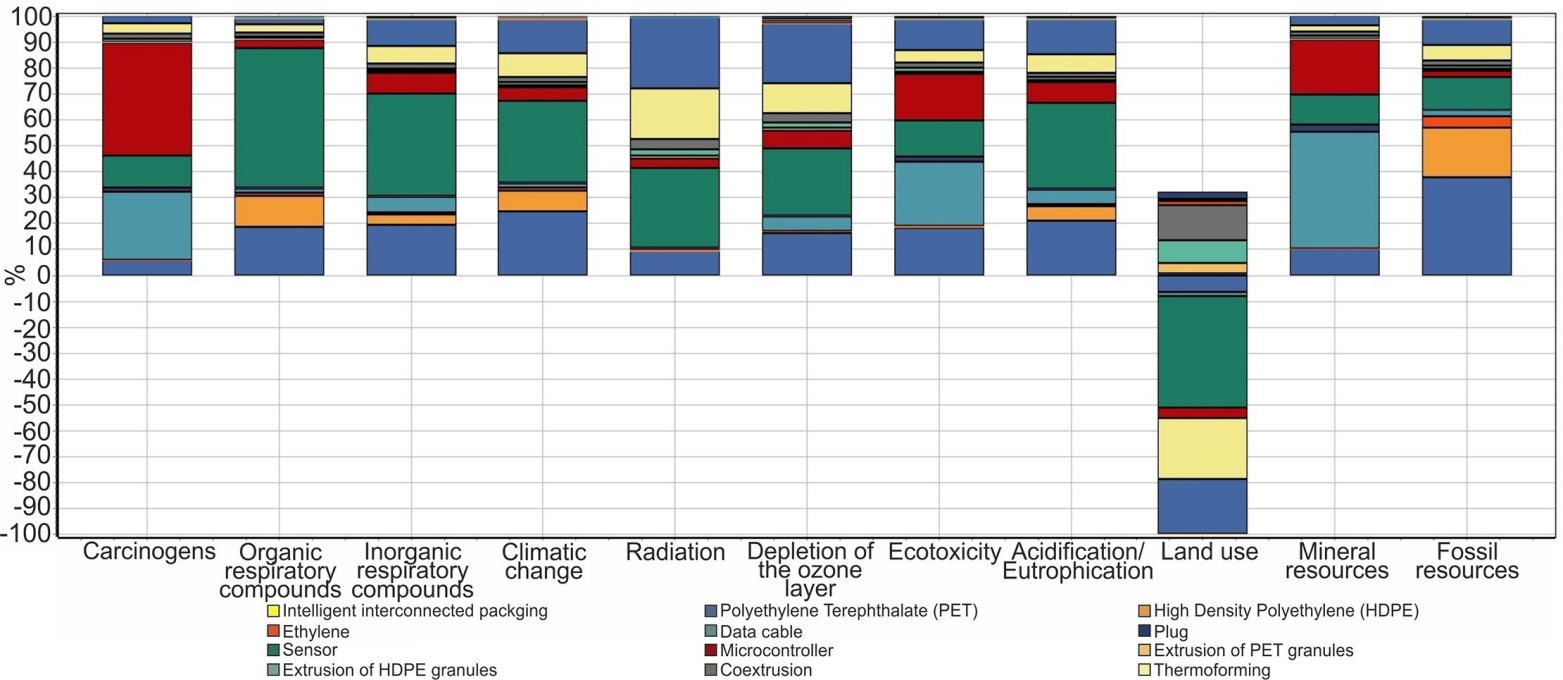
Results of the characterization of LCA for intelligent interconnected packaging in the SimaPro software.

It is clear that the greatest environmental impact in most categories is led by the intelligent system components (Fig 3).

### Impact assessment by standardized category

**Fig 4:**
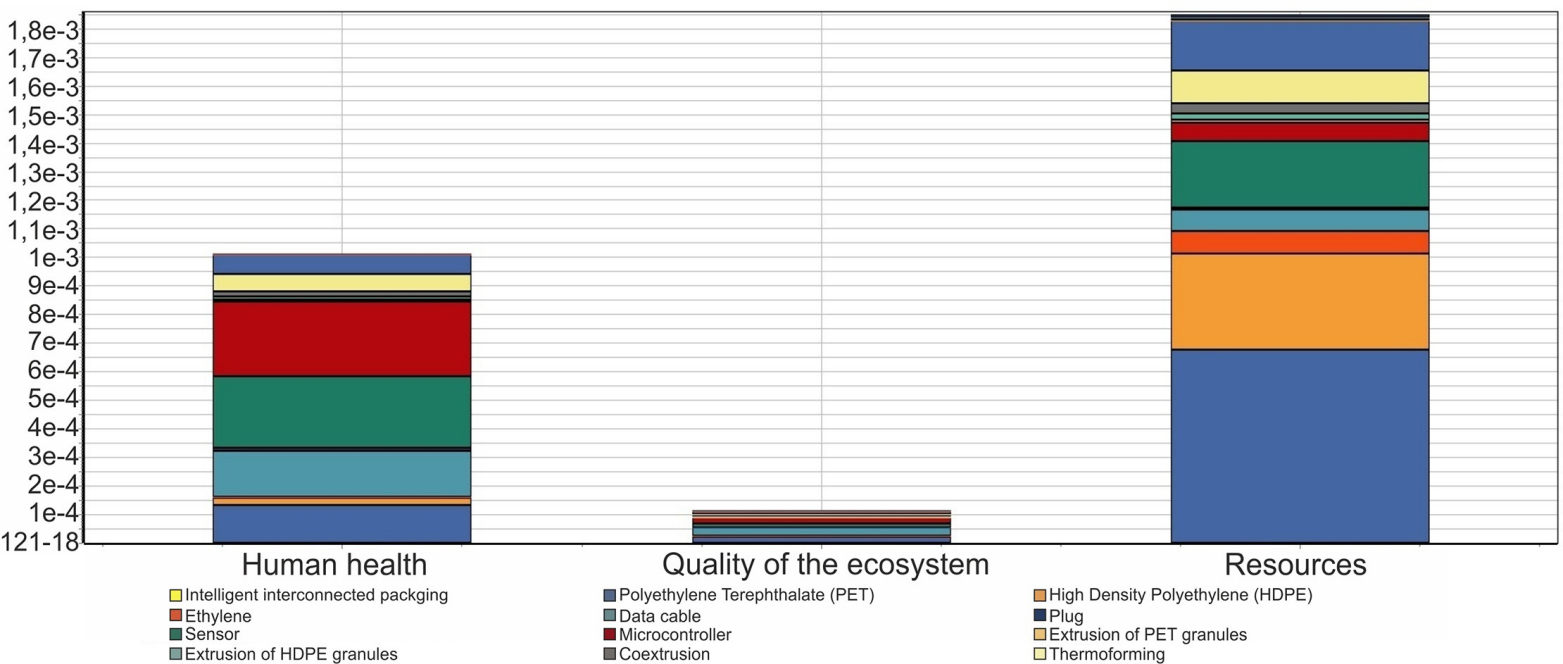
Standardized impact values in the different categories of the LCA for smart interconnected packaging in the SimaPro software.

First, the category of greatest impact is that of the consumption of resources, and is led by the chosen plastics (PET and HDPE). This impact arises from the fact that they are non-renewable raw materials (Fig 4).

This is followed by the category of human well-being, where there is a high impact of the electronic components of the intelligent system (microcontroller, sensor, and cable). The third and last category of impact involves the quality of the ecosystem, which lies far below the other two aforementioned categories.

### Single score per component

**Fig 5:**
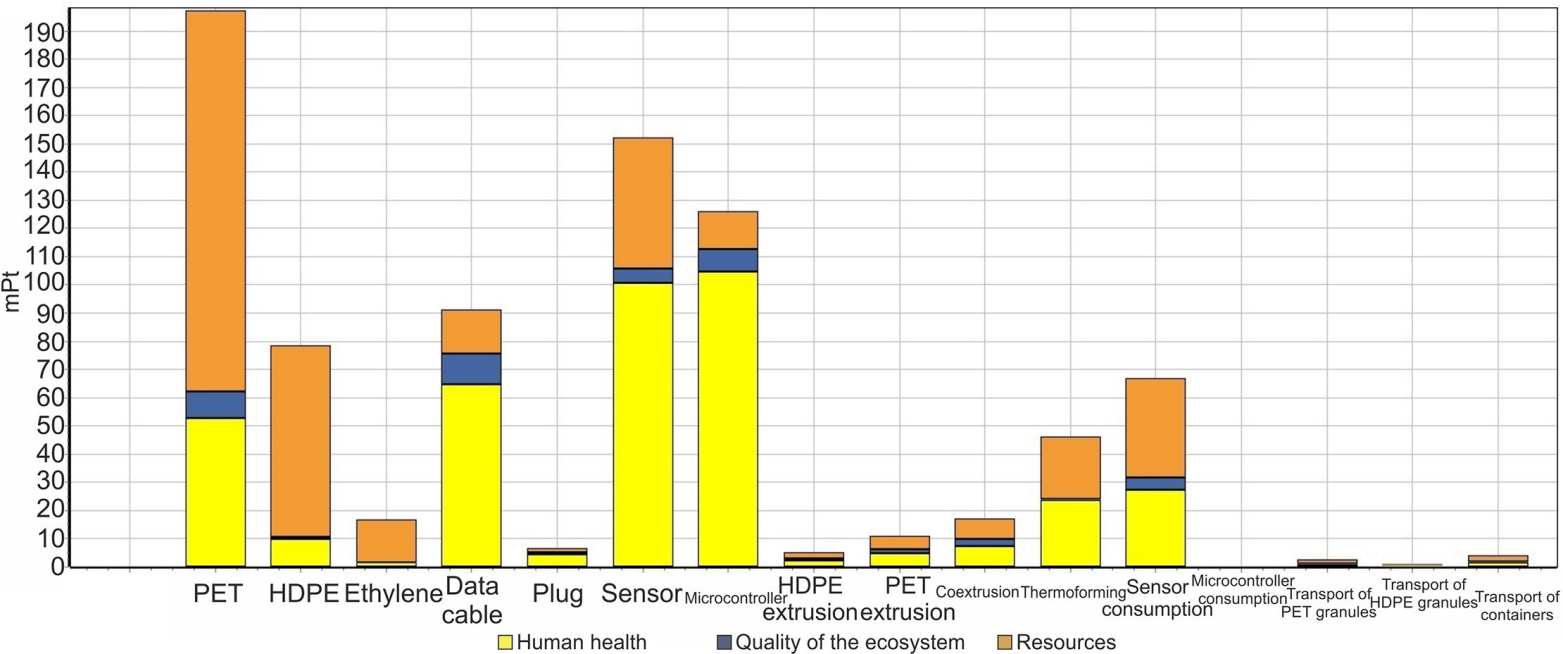
Single score per component for the various components of the smart interconnected packaging in the SimaPro software.

The greatest total impact is observed to be caused by PET, which is closely followed by that of the sensor, and then by the microcontroller (Fig 5).

## Discussion

### Basic discrepancies

The basic discrepancies between these two systems can be summarized in the following sentence: 'The aim is to measure a qualitative plan to create a beneficial future footprint through the use of a quantitative instrument designed to measure an existing harmful environmental footprint', which can be refuted by analysing each concept individually.

The degree to which the defined qualities of a C2C product can be measured with an LCA depends on what percentage can be quantified for the measurement. In other words, to what extent the qualities can be described in specific terms rather than just conceptually. On the other hand, once a future scenario has been agreed, including backcasting (a tool for planning long-term projects) and intermediate milestones, it is possible to evaluate future technologies using an LCA. That is, the 'C2C-ness' of the products can be measured if they are defined using data that reflects the probable nature of the future C2C design solution. However, the measurability of this data depends on the ability to accurately predict future results.

In addition, although the LCA has been designed to measure the harmful (negative) environmental impact of the products, it can also be used to measure the established beneficial (positive) footprint of a C2C product. This would not be used to calculate 'damages', but to calculate the benefits of the 'solutions' (the evaluation of ideas) or, in other words, the degree to which the solutions contribute to the declared intentions [48].

### Measurement of C2C principles with LCA

Principle 1: Waste equals food (Garbage = Food)

Definition of useful life of the products: in the eco-design, there are no strict rules to define the useful life of a product and, as a general rule, long useful lives are usually preferred. On the other hand, C2C works with end-of-use scenarios and not with end-of-life scenarios [48].

Interaction of technical and biological metabolisms: according to the C2C concept, neither of the two corresponding metabolisms has a higher priority than the other. However, through an LCA, it is possible to evaluate the most sustainable disposal option [37].

Effects of adding nutrients to the environment: according to the Cradle to Cradle concept, adding biological nutrients to the environment is always a good thing. This claim is based on the fact that many parts of the environment lack nutrients due to human activities and, therefore, need the addition of biological nutrients to regain their natural balance. On the other hand, an LCA could clearly show that, for example, the incineration of paper for electricity production is preferable to composting [37].

Recycling concept: in C2C it is stated that materials improve quality through recycling as a result of design decisions before starting production. Under LCA, the concept of recycling is not necessarily positive. The benefit of recycling only manifests itself when the recycled material is used to replace the virgin material [48].

Toxicity: C2C measures the evaluation of the possibility of living systems coming into contact (oral, dermal, by inhalation) with substances/materials during the past, present, and foreseen future of the product using the ABC-X method, which determines and classifies the quality of the nutrients. In LCA, the toxicity calculation is made on the basis of the real impact that occurs as a result of the release of toxic substances (emissions), assuming that some of the chemical products contained in said toxic substances are emitted [48].

Principle 2: Use of sustainable energy

Cradle 2 Cradle considers how energy is produced and how effectively it is used, while LCA considers the amount of energy used throughout the life of the product.

It is possible to calculate an LCA while considering only solar energy as the energy input. However, this would be carried out based on the current technology of solar panels, and hence this calculation should be corrected in the future to also consider the most efficient future technology that is expected to be developed [48].

Principle 3: Celebrate diversity

Regarding this principle, in recent years, around the union of the concepts of LCA and Triple E (Sustainability), the first studies have arisen where the Social Life Cycle Assessment (SLCA) is carried out. This SLCA considers the welfare of members belonging to the production chain [7,49].

### Application of the C2C principles to the case study

Principle 1: Since the product cannot be recycled because it is bilayer, compliance with this principle is proposed by studying the possibility of incineration with energy recovery, so that this energy constitutes the same process or an entry for another product. In addition, with respect to the materials used, the ABC-X evaluation methodology of the C2C is proposed as a basis, since it is one of its strong points, and, for the present package, high values are observed at that point. It should be borne in mind that, as part of the eco-design (fundamental pillar of the C2C), the container can be dismantled, which facilitates its disposal at the end of its useful life, whereby its components can be divided into the pre-established cycles.

Principle 2: A study is proposed into how values obtained would change if wind, water, and/or solar energy were used instead of electrical energy, for example. The purchase of renewable energy credits is also evaluated. With regard to energy efficiency, the activity time of the sensors could be optimized through their study. In the present work, the chosen microcontrollers have a saving mode, which greatly alleviates the load of electrical consumption of the intelligent system. The option of evaluating the use of conductive polymer sensors is also suggested, since their operating temperature is low.

Principle 3: All raw material suppliers are assumed to be Spanish, and they therefore contribute to the increase of local employment, in addition to the establishment of the production plant in Spain, thus generating opportunities for training and employment.

Please note that the recommendation to use only Spanish suppliers is specific to the case study considered, as a result of the Social Life Cycle Assessment (SLCA) that has been carried out. The generalizable recommendations should be to celebrate diversity, as introduced in previous sections, and develop a SLCA considering the welfare of members belonging to the production chain.

### Process optimization

To begin with, the reduction of the thickness of the plastics used is first considered, which would not cause any change in the production line, because no different industrial machinery would be required, and the amount of raw material transported and used for the manufacture of the film would be reduced (30). To this end, specific laboratory tests must be developed in order to verify the technical feasibility of the evaluation solution, in particular, the possible changes that occur in the permeability of the film and the mechanical behaviour after the thickness of the film is reduced.

A second option is to use recycled plastic granules, which would increase the level of environmental sustainability associated with the life cycle of the container, although it may turn out to be less effective compared to reducing the film thickness since the recycling of plastic waste and the transport of recycled granules to the factory imply a high consumption of electricity [30].

The purification and reuse of the intrinsic waste obtained in the extrusion of granules is also proposed.

On the other hand, the supply chain could be optimized, 1by evaluating the distribution through alternative transport systems and via then optimization at the potential location site of the production plant.

The evaluation of the passage from a bilayer container to a monolayer container is considered, thereby extending its end-of-life options, and recycling is subsequently contemplated, which, in principle, would contribute towards reducing the environmental impact. This process should be analysed meticulously, since it is not easy to ascertain all the properties of a multilayer container gives through the application of a single component. Parameters, such as the thickness and the amount of material to be used, must inevitably be modified.

Finally, the reduction of the weight of the container is considered since it weighs almost 3.5Kg; this value is very high for its transport, and hence the "weight" parameter must be considered as a major factor when choosing the plastic.

## Conclusions

It is concluded that smart packaging systems provide a viable option for the improvement of the minimization of food wastage, which has become one of the main problems currently facing the food industry.

Tools for the evaluation of sustainability, such as Life Cycle Assessment, can help assess the environmental impact of the proposed solution.

In addition, this study seeks to encourage product designers to carry out their work in a more sustainable manner, by following eco-design criteria when launching projects. The necessary tools are provided and special emphasis is placed on the follow-up of the product 'from cradle to cradle'.

It is noted that the choice of smart packaging technology and packaging characteristics in general (chosen material, thickness, dimensions) depend intrinsically on the type of food to be controlled, whose properties and behaviour must be meticulously studied in order to find the optimal packaging solution for each specific type of food.

On the other hand, unlike other Life Cycle Assessment processes observed in the literature referring to non-intelligent packaging, the transport phase does not imply a high environmental impact. This shows that by adding a smart system to the packaging, the environmental load is relocated, passing from the transport phase to the intelligent system.

Two ways to continue with the present investigation are considered:

The sensor of ethylene detection can be extended to that of an electronic nose, since aging and bacterial putrefaction of the food generate complex aromas, formed by the presence of various volatile organic compounds, which could be detected by this system.

As a second option, it is suggested that the packaging should be expanded to several versions specialised for other specific types of food, thus constituting a "shopping cart", thanks to the existence of electronic noses that can detect the degradation compounds of these types of food, including meat, oil, eggs, and milk.
