# Correction: Aspects of sustainability and design engineering for the production of interconnected smart food packaging

**DOI:** 10.1371/journal.pone.0218337

**Published:** 2019-06-06

**Authors:** María Inés Cabot, Amalia Luque, Ana de las Heras, Francisco Aguayo

## Notice of republication

An incorrect version of Fig 2 was published in error. This article was republished on May 31, 2019 to correct for this error. In addition, an updated Funding statement is included in the republished article. Please download this article again to view the correct version.
